# Longitudinal study of the relationship between number of prior miscarriages or stillbirths and changes in quality of life of pregnant women: the Japan Environment and Children’s Study (JECS)

**DOI:** 10.1186/s12884-023-05578-6

**Published:** 2023-04-28

**Authors:** Kaori Futakawa, Kenta Matsumura, Akiko Tsuchida, Mizuho Konishi, Hatoko Sasaki, Hidetoshi Mezawa, Kiwako Yamamoto–Hanada, Hidekuni Inadera, Tomomi Hasegawa, the Japan Environment and Children’s Study (JECS) Group, Michihiro Kamijima, Shin Yamazaki, Yukihiro Ohya, Reiko Kishi, Nobuo Yaegashi, Koichi Hashimoto, Chisato Mori, Shuichi Ito, Zentaro Yamagata, Takeo Nakayama, Tomotaka Sobue, Masayuki Shima, Hiroshige Nakamura, Narufumi Suganuma, Koichi Kusuhara, Takahiko Katoh

**Affiliations:** 1 Department of Maternal Nursing, Faculty of Medicine, Academic Assembly, University of Toyama, 2630 Sugitani, Toyama-Shi, Toyama 930-0194 Japan; 2 Department of Public Health, Faculty of Medicine, University of Toyama, 2630 Sugitani, Toyama, Toyama-Shi, Toyama 930-0194 Japan; 3 Toyama Regional Center for JECS, University of Toyama, 2630 Sugitani, Toyama-Shi, Toyama 930-0194 Japan; 4 Department of Psychology, Tokyo Seitoku University, 1-7-13 Jujodai, Kita-Ku, Tokyo, 114-0033 Japan; 5 Medical Support Center for Japan Environment and Children’s Study (JECS), National Center for Child Health and Development, 2-10-1 Okura, Setagaya-Ku, Tokyo, 157-8535 Japan; 6 Shizuoka Graduate University of Public Health, 4-27-2, Kita-Ando, Aoi-Ku, Shizuoka-Shi, Shizuoka, 420-0881 Japan

**Keywords:** Miscarriage, Stillbirth, Quality of life, Pregnant women, Longitudinal study

## Abstract

**Background:**

Although a history of miscarriage or stillbirth has been reported to negatively affect quality of life (QOL) during the subsequent pregnancy, the association between the number of previous miscarriages or stillbirths and QOL, as well as trends in QOL during pregnancy, has not been clarified. This study sought to determine this association during early and mid- to late pregnancy.

**Methods:**

Data from 82,013 pregnant women who participated in the Japan Environment and Children’s Study (JECS) from January 2011 to March 2014 were analyzed. In early and mid/late pregnancy, participants completed questionnaires and QOL was assessed using the Physical and Mental Component Summary (PCS and MCS, respectively) scores from the 8-item Short-Form Health Survey (SF-8). The pregnant women were divided into four groups according to number of previous miscarriages or stillbirths (0, 1, 2, and ≥ 3), and the PCS and MCS scores in early pregnancy and mid/late pregnancy were compared between group 0 and groups 1, 2, and ≥ 3. Generalized linear mixed models were used for analysis.

**Results:**

PCS score in early pregnancy was lower in group 1 (β =  − 0.29, 95% confidence interval [CI] − 0.42 to − 0.15), group 2 (β =  − 0.45, 95% CI − 0.73 to − 0.18), and group ≥ 3 (β =  − 0.87, 95% CI − 1.39 to − 0.35) than in group 0. Group 1 and group ≥ 3 showed a trend for increased PCS score during pregnancy (β = 0.22, 95% CI 0.07 to 0.37 and β = 0.75, 95% CI 0.18 to 1.33, respectively) compared with group 0.

**Conclusions:**

PCS score in early pregnancy was lower with a more frequent history of miscarriage or stillbirth. However, in terms of changes in QOL during pregnancy, pregnant women with a history of miscarriage or stillbirth showed greater increases in PCS score during mid/late pregnancy than pregnant women with no history of miscarriage or stillbirth.

**Supplementary Information:**

The online version contains supplementary material available at 10.1186/s12884-023-05578-6.

## Background

Miscarriage is the early termination of pregnancy and the definition depends on the country and academic organization. The American College of Obstetricians & Gynecologist (ACOG) [1] defines the pregnancy loss at 13 weeks of gestation, the European Society of Human Reproduction and Embryology (ESHRE) [2] at 24 weeks, and the Japan Society of Obstetrics and Gynecology at 22 weeks [3]. The frequency of miscarriage is estimated to be 10–15% of all pregnancies [2]. If two or three or more repeated miscarriages occur, recurrent pregnancy loss (RPL) [1, 2, 4] is diagnosed, and its frequency is said to be 0.8–2% [5, 6]. The incidence of stillbirth is 0.6% to 1.8% [7, 8].

Previous studies comparing pregnant women with a history of miscarriage or stillbirth with those without such history have reported that those with a history have more distress specific to the early and late stages of pregnancy [9] or strong depression and anxiety [10–12]. In cases of RPL, physical and emotional recovery is even lengthier. Surveys of nonpregnant women with RPL reported that 33.0% were depressed [13] and that 15.4% had depression or anxiety disorders [14]. In addition, previous studies of the effects of RPL on women’s subsequent pregnancy have found that depression and anxiety are stronger in early pregnancy [15, 16]. In addition, depression in early pregnancy was reported to decrease the quality of life (QOL) of pregnant women [17] and, because pregnant women with a history of miscarriage or stillbirth are more depressed in early pregnancy, this may affect their QOL in the subsequent pregnancy.

Indeed, research on the impact of a history of miscarriage or stillbirth on QOL during the subsequent pregnancy found that pregnant women with such history do have a lower QOL than those without such history [18, 19]. However, the association between the number of previous miscarriages or stillbirths and QOL during the subsequent pregnancy has not been investigated. In addition, cross-sectional studies have mainly been conducted at a single time point during pregnancy, and no studies have examined QOL trends longitudinally. Thus, the impact of the number of previous miscarriages or stillbirths on QOL during the subsequent pregnancy has not been clarified.

To this end, using data obtained in a large nationwide cohort study, we divided pregnant women with a history of miscarriages or stillbirths into three groups according to the number of previous miscarriages or stillbirths and compared their QOL in early pregnancy and the change in their QOL from early to mid- to late pregnancy with the QOL of pregnant women who had no history of miscarriages and stillbirths.

## Methods

### Study design and population

**Fig. 1:**
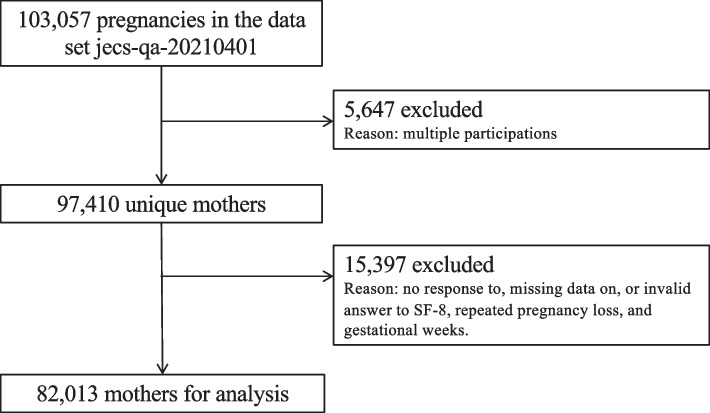
Participant flow diagram

The study design was a prospective cohort study. For this birth cohort study, the dataset for analysis comprised data from pregnant women who participated in the Japan Environment and Children’s Study (JECS), the protocol of which has been described in detail elsewhere [20, 21]. Briefly, JECS recruitment took place between January 2011 and March 2014, and participants (mothers, fathers, and their newborn babies) were recruited and followed at 15 regional centers across Japan. In the present study, from a total of 103,057 pregnancies included in the jecs-qa-20210401 dataset (released in April 2021), 5,647 pregnant women with multiple participations were excluded. Furthermore, pregnant women who did not complete the 8-item Short-Form Health Survey (SF-8) or had missing data on the number of miscarriages or stillbirths or on gestational weeks (*n* = 15,397) were excluded, leaving a total of 82,013 pregnant women for analysis in this study (Fig. 1).

The JECS protocol was reviewed and approved by the Ministry of the Environment’s Institutional Review Board on Epidemiological Studies and the Ethics Committees of all participating institutions. Written informed consent for participation was obtained from all participants. The protocol of the present study was also approved by the Ethics Committee of the University of Toyama.

### Data collection

The pregnant women completed the JECS questionnaire twice, once in early pregnancy and once in mid/late pregnancy, when we could contact them directly. The questionnaire in early pregnancy included items on demographics (e.g., occupation, marital status, family structure, smoking habits, drinking habits, diet, and physical activity), medical history (e.g., cardiac disease, gastrointestinal disease, urological disease, allergic disease, autoimmune disease, endocrine disease, and gynecological disease), and obstetric history. In addition to the above, the questionnaire for pregnant women in mid/late pregnancy asked about educational background and household income. Data were also obtained from transcripts from the medical records kept by physicians, midwives/nurses, and/or research coordinators in early pregnancy (e.g., maternal age, height, weight, and details of previous pregnancies [method of delivery, complications, miscarriage, stillbirth, and abortion]).

### Measures

#### Exposure

Based on a previous study [22] that found that the greater the number of miscarriages, the greater the emotional impact of miscarriage and the more negative and painful the impact, the pregnant women who participated in the study were divided into four groups according to the number of miscarriages or stillbirths they had experienced: 0, 1, 2, or ≥ 3. In this study, miscarriage was defined as spontaneous abortion before 22 weeks of gestation and stillbirth as fetal death after 22 weeks.

#### Outcome

The Japanese version of the SF-8 [23] was used to assess the QOL of the pregnant women. The SF-8 is a shortened version [24] of the Medical Outcomes Study 36-item Short-Form (SF-36) [25] and comprises eight questions corresponding to the eight lower dimensions of the SF-36: general health (GH) asks about respondent’ overall health; physical functioning (PF) asks the extent to which physical reasons prevented them from doing their usual daily physical activities; role physical (RP) asks the extent to which physical reasons prevented them from doing their usual daily work; bodily pain (BP) asks how much pain they have experienced in their body; vitality (VT) asks the extent of their vitality;, social functioning (SF) asks the extent to which physical or emotional reasons prevented them from socializing with family and friends; mental health (MH) asks how much they have suffered from psychological problems; and role emotional (RE) asks the extent to which emotional reasons prevented them from doing their daily activities. The Physical Component Summary (PCS) and Mental Component Summary (MCS) scores are calculated from the scores of the lower eight dimensions, with the PCS score representing physical health (mainly explained by GH, PF, RP, and BP) and the MCS score representing mental health (mainly explained by VT, SF, MH, and RE). The PCS and MCS scores are calculated using weighting coefficients, which are set so that the mean score of Japanese nationals is 50 each for the PCS and MCS and the standard deviation is 10. This scale indicates that higher PCS and MCS scores indicate higher QOL. The reliability and validity of the Japanese version of the SF-8 have been confirmed through comparisons with the SF-36 and via factor analysis [24].

#### Covariates

The covariates were as follows: age during pregnancy; pre-pregnancy BMI; parity (primiparous or multiparous); physical activity; history of depression, anxiety disorder, dysautonomia, or schizophrenia (no or yes); history of any physical disease (no or yes); marital status (married, single, divorced or widowed); employed during early pregnancy (no or yes); highest educational level (≤ 12, 12 to < 16, ≥ 16 years); annual household income (< 4, 4 to < 6, ≥ 6 million JPY); alcohol intake (never, former, or current); smoking status (never, quit before realizing of current pregnancy, quit after realizing of current pregnancy, or current smoker); morning sickness (never, nausea but no vomiting, vomiting but able to eat, vomiting and unable to eat); questionnaires administered in early pregnancy; and questionnaires administered in mid/late pregnancy. All covariates are categorized according to standard medical practice or common practice in Japan [26]. Missing data were also included in the model as dummy-coded variables.

### Data analysis

The PCS and MCS scores of women in early pregnancy were compared with the scores in mid/late pregnancy. Then, the PCS and MCS scores of pregnant women in early pregnancy were compared between the three groups with a history of miscarriages or stillbirths (1, 2, or ≥ 3) and the pregnant women who had no history of miscarriages and stillbirths. Also, changes in PCS and MCS scores from early pregnancy to mid/late pregnancy were then compared between the three groups and the pregnant women with no history of miscarriage and stillbirth. The SF-8 subscale scores in early pregnancy were then compared between the three groups and the pregnant women who had no history of miscarriages and stillbirths. Generalized linear mixed models were used for these analyses. In the adjusted models, all variables in the background table and their interactions with assessment timing were used with forced entry methods.

In addition, participants were divided into primiparas and multiparas, and similar analyses were performed.

All analyses were performed using SAS version 9.4 software (SAS Institute Inc.). Statistical significance was set at 5%.

## Results

### Characteristics of the pregnant women

Table 1 shows the demographic and obstetric characteristics of the pregnant women. The number of primiparas was 36,557 (44.6%) and the number of multiparas was 45,456 (55.4%). More than 94% of the pregnant women were married and more than 54% were employed.

**Table 1:** Characteristics of pregnant women according to history of pregnancy loss (*N* = 82,013)

	Number of miscarriages or stillbirths			
	0	1	2	≥ 3
	(*n* = 66,044)	(*n* = 12,568)	(*n* = 2,678)	(*n* = 723)
	n (%)	n (%)	n (%)	n (%)
Age, y				
Median [IQR]	30 [27, 34]	32 [29, 36]	34 [30, 37]	35 [32, 38]
Missing, n	10	1	1	0
Pre-pregnancy body mass index, kg/m^2^				
Median [IQR]	20.5 [19.1, 22.4]	20.7 [19.2, 22.7]	20.8 [19.3, 23.0]	20.8 [19.3, 22.9]
Missing, n	29	3	1	0
Physical activity, METs･h/day				
Median [IQR]	2.1 [0.5, 7.3]	1.9 [0.5, 6.6]	1.9 [0.5, 6.6]	1.9 [0.5, 5.8]
Missing, n	996	192	32	14
Live birth experience				
No	31,403 (47.6)	4,260 (33.9)	703 (26.3)	191 (26.4)
Yes	34,641 (52.5)	8,308 (66.1)	1,975 (73.8)	532 (73.6)
History of depression, anxiety disorder, dysautonomia, or schizophrenia				
No	56,656 (85.8)	10,703 (85.2)	2,274 (84.9)	597 (82.6)
Yes	9,388 (14.2)	1,865 (14.8)	404 (15.1)	126 (17.4)
History of any physical disease				
No	11,572 (17.5)	1,911 (15.2)	402 (15.0)	92 (12.7)
Yes	54,472 (82.5)	10,657 (84.8)	2,276 (85.0)	631 (87.3)
Marital status				
Married	62,607 (94.8)	12,190 (97.0)	2,598 (97.0)	699 (96.7)
Single	2,689 (4.1)	205 (1.6)	22 (0.8)	3 (0.4)
Divorced or widowed	473 (0.7)	132 (1.1)	45 (1.7)	20 (2.8)
Missing	275 (0.4)	41 (0.3)	13 (0.5)	1 (0.1)
Employed during early pregnancy				
No	22,748 (34.4)	4,825 (38.4)	1,119 (41.8)	298 (41.2)
Yes	41,242 (62.5)	7,305 (58.1)	1,461 (54.6)	393 (54.4)
Missing	2,054 (3.1)	438 (3.5)	98 (3.7)	32 (4.4)
Highest education level, y				
≤ 12	23,261 (35.2)	4,450 (35.4)	1,010 (37.7)	277 (38.3)
12 to < 16	27,807 (42.1)	5,473 (43.6)	1,140 (42.6)	317 (43.9)
≥ 16	14,722 (22.3)	2,598 (20.7)	519 (19.4)	125 (17.3)
Missing	254 (0.4)	47 (0.4)	9 (0.3)	4 (0.6)
Annual household income, million JPY				
< 4	24,867 (37.7)	4,472 (35.6)	923 (34.5)	239 (33.1)
4 to < 6	20,401 (30.9)	4,004 (31.9)	816 (30.5)	231 (32.0)
≥ 6	16,359 (24.8)	3,340 (26.6)	766 (28.6)	210 (29.1)
Missing	4,417 (6.7)	752 (6.0)	173 (6.5)	43 (6.0)
Alcohol intake				
Never	22,736 (34.4)	4,366 (34.7)	927 (34.6)	246 (34.0)
Former	36,689 (55.6)	6,830 (54.3)	1,403 (52.4)	393 (54.4)
Current	6,378 (9.7)	1,330 (10.6)	336 (12.6)	84 (11.6)
Missing	241 (0.4)	42 (0.3)	12 (0.5)	0 (0.0)
Smoking history				
Never	39,066 (59.2)	7,082 (56.4)	1,453 (54.3)	388 (53.7)
Quit before realizing of current pregnancy	14,842 (22.5)	3,295 (26.2)	698 (26.1)	220 (30.4)
Quit after realizing of current pregnancy	8,854 (13.4)	1,502 (12.0)	349 (13.0)	78 (10.8)
Current smoker	2,889 (4.4)	610 (4.9)	168 (6.3)	31 (4.3)
Missing	393 (0.6)	79 (0.6)	10 (0.4)	6 (0.8)
Morning sickness				
Never	11,650 (17.6)	1,823 (14.5)	331 (12.4)	86 (11.9)
Nausea but no vomiting	27,976 (42.4)	5,646 (44.9)	1,241 (46.3)	324 (44.8)
Vomiting but able to eat	18,982 (28.7)	3,691 (29.4)	804 (30.0)	229 (31.7)
Vomiting and unable to eat	7,211 (10.9)	1,366 (10.9)	292 (10.9)	84 (11.6)
Missing	225 (0.3)	42 (0.3)	10 (0.4)	0 (0.0)
Questionnaires administered in early pregnancy				
Median [IQR]	15 [12, 18]	14 [12, 17]	15 [12, 17]	15 [12, 18]
Questionnaires administered in mid/late pregnancy				
Median [IQR]	27 [25, 29]	27 [25, 29]	27 [25, 29]	27 [25, 29]

Overall, 66,044 (80.5%) pregnant women had no history of miscarriage or stillbirth, 12,568 (15.3%) had a history of 1 miscarriage or stillbirth, 2,678 (3.3%) had a history of 2 miscarriages or stillbirths, and 723 (0.9%) had a history of ≥ 3 miscarriages or stillbirths.

### Changes in PCS and MCS scores for all pregnant women from early pregnancy to mid/late pregnancy

Table 2 shows the results of the generalized linear mixed model. Both the PCS score (β = 0.66, 95% CI 0.40 to 0.92, *p* < 0.0001) and MCS score (β = 2.20, 95% CI 1.95 to 2.45, *p* < 0.0001) increased significantly during pregnancy.

**Table 2:** Results of linear mixed model analyses of PCS and MCS scores for pregnant women

Effect	Level	Period	Crude		Adjusted^a^	
	NMS		PCS	MCS	PCS	MCS
			β (95%CI)	β (95%CI)	β (95%CI)	β (95%CI)
Period		Early	reference	reference	reference	reference
		Mid/late	**0.66 (0.60, 0.72)**	**3.05 (3.00, 3.11)**	**0.66 (0.40, 0.92)**	**2.20 (1.95, 2.45)**
NMS	0	Early	45.13 (45.07, 45.18)	46.05 (45.99, 46.10)	reference	reference
	1	Early	** − 0.51 (− 0.65, − 0.37)**	− 0.06 (− 0.20, 0.08)	** − 0.29 (− 0.42, − 0.15)**	− 0.12 (− 0.25, 0.02)
	2	Early	** − 0.78 (− 1.07, − 0.49)**	− 0.10 (− 0.38, 0.18)	** − 0.45 (− 0.73, − 0.18)**	− 0.15 (− 0.42, 0.13)
	≥ 3	Early	** − 1.42 (− 1.97, − 0.86)**	− 0.16 (− 0.71, 0.40)	** − 0.87 (− 1.39, − 0.35)**	− 0.14 (− 0.66, 0.39)
NMS × Period	0 ×	Mid/late	reference	reference	reference	reference
	1 ×	Mid/late	**0.19 (0.04, 0.35)**	0.08 (− 0.06, 0.23)	**0.22 (0.07, 0.37)**	0.12 (− 0.03, 0.26)
	2 ×	Mid/late	0.17 (− 0.15, 0.49)	− 0.15 (− 0.45, 0.15)	0.21(− 0.01, 0.52)	− 0.08 (− 0.38, 0.21)
	≥ 3 ×	Mid/late	**0.81 (0.21, 1.41)**	− 0.06 (− 0.67, 0.55)	**0.75 (0.18, 1.33)**	− 0.01 (− 0.61, 0.59)

Boldface indicates significance at *p* values less than 0.05

*NMS* Number of miscarriages or stillbirths, *PCS* Physical Component Summary, *MCS* Mental Component Summary

^a^Adjusted for age during pregnancy; pre-pregnancy BMI; parity; physical activity; history of depression, anxiety disorder, dysautonomia, or schizophrenia; history of any physical disease; marital status; employed during early pregnancy; highest educational level; annual household income; alcohol intake; smoking status; morning sickness; questionnaires administered in early pregnancy; questionnaires administered in mid/late pregnancy

### Differences in PCS and MCS scores in early pregnancy by number of miscarriages or stillbirths

As shown in Table 2, PCS scores in early pregnancy were significantly lower in the groups with a history of 1 miscarriage or stillbirth (mean PCS = 45.39, β =  − 0.29, 95% CI − 0.42 to − 0.15, *p* < 0.0001), 2 miscarriages or stillbirths (mean PCS = 45.23, β =  − 0.45, 95% CI − 0.73 to − 0.18, *p* = 0.0014), and ≥ 3 miscarriages or stillbirths (mean PCS = 44.81, β =  − 0.87, 95% CI − 1.39 to − 0.35, *p* = 0.0011) compared with the group with no history of miscarriage or stillbirth (mean PCS score = 45.68). The higher the number of miscarriages and stillbirths, the lower the PCS score. There was no significant difference in their MCS score according to the number of miscarriages and stillbirths.

### Changes in PCS and MCS scores from early pregnancy to mid/late pregnancy by number of miscarriages or stillbirths

Also shown in Table 2, the group with a history of 1 miscarriage or stillbirth and the group with ≥ 3 miscarriages or stillbirths showed a significantly positive association with PCS score (β = 0.22, 95% CI 0.07 to 0.37, *p* = 0.0043 and β = 0.75, 95% CI 0.18 to 1.33, *p* = 0.0104, respectively) compared with the group with no history.

### Differences in SF-8 subscale scores by number of miscarriages or stillbirths

Compared with the group without a history of miscarriage or stillbirth, the three groups with a history of miscarriage or stillbirth had significantly lower scores for the PF, RP, and SF of the eight SF-8 subscales in early pregnancy (Table 3). The higher the number of miscarriages and stillbirths, the lower the PF, RP, and SF scores.

**Table 3:** Results of linear mixed model analyses of SF-8 subscale scores for pregnant women

Effect	Level	Period	Adjusted^a^							
	NMS		General health	Physical functioning	Role physical	Bodily pain	Vitality	Social functioning	Mental health	Role emotional
			β(95%CI)	β (95%CI)	β (95%CI)	β (95%CI)	β (95%CI)	β (95%CI)	β (95%CI)	β (95%CI)
Period		Early	reference	reference	reference	reference	reference	reference	reference	reference
		Mid/late	**4.56** **(4.29, 4.82)**	** − 0.48** **(− 0.76, − 0.20)**	**2.81** **(2.48, 3.13)**	** − 3.31** **(− 3.61, -3.00)**	**3.37** **(3.11, 3.63)**	**2.11** **(1.78, 2.45)**	**0.51** **(0.27, 0.75)**	**1.63** **(1.34, 1.91)**
NMS	0	Early	reference	reference	reference	reference	reference	reference	reference	reference
	1	Early	0.10 (− 0.03, 0.24)	** − 0.43** **(− 0.57, − 0.28)**	** − 0.51** **(− 0.68, − 0.34)**	− 0.06 (− 0.21, 0.10)	− 0.09 (− 0.22, 0.05)	** − 0.41** **(− 0.59, − 0.24)**	** − 0.18** **(− 0.31, − 0.05)**	− 0.15 (− 0.30, 0.01)
	2	Early	0.10 (− 0.17, 0.38)	** − 0.73** **(− 1.03, − 0.42)**	** − 0.75** **(− 1.10, − 0.39)**	− 0.10 (− 0.41, 0.20)	− 0.02 (− 0.29, 0.26)	** − 0.57** **(− 0.92, − 0.21)**	** − 0.34** **(− 0.60, − 0.08)**	− 0.18 (− 0.50, 0.13)
	≥ 3	Early	0.14 (− 0.38, 0.67)	** − 1.12** **(− 1.71, − 0.53)**	** − 1.11** **(− 1.80, − 0.42)**	− 0.51 (− 1.10, 0.09)	− 0.01 (− 0.51, 0.50)	** − 0.95** **(− 1.63, − 0.28)**	− 0.07 (− 0.56, 0.41)	** − 0.75** **(− 1.40, − 0.10)**
NMS × Period	0 ×	Mid/late	reference	reference	reference	reference	reference	reference	reference	reference
	1 ×	Mid/late	0.03 (− 0.12, 0.18)	**0.27** **(0.11, 0.44)**	**0.41** **(0.22, 0.59)**	− 0.01 (− 0.18, 0.16)	**0.21** **(0.06, 0.36)**	**0.22** **(0.02, 0.41)**	**0.14** **(0.00, 0.28)**	0.15 (− 0.01, 0.32)
	2 ×	Mid/late	− 0.16 (− 0.46, 0.15)	**0.38** **(0.03, 0.73)**	0.16 (− 0.23, 0.56)	0.15 (− 0.18, 0.49)	0.01 (− 0.29, 0.31)	0.11 (− 0.29, 0.51)	0.16 (− 0.12, 0.44)	− 0.19 (− 0.54, 0.17)
	≥ 3 ×	Mid/late	0.07 (− 0.50, 0.63)	**0.93** **(0.26, 1.59)**	0.64 (− 0.11, 1.40)	**0.87** **(0.23, 1.51)**	− 0.13 (− 0.66, 0.40)	0.22 (− 0.55, 0.98)	0.27 (− 0.25, 0.79)	0.54 (− 0.21, 1.28)

Boldface indicates significance at *p* values less than 0.05

*NMS* Number of miscarriages or stillbirths

^a^Adjusted for age during pregnancy; pre-pregnancy BMI; parity; physical activity; history of depression, anxiety disorder, dysautonomia, or schizophrenia; history of any physical disease; marital status; employed during early pregnancy; highest educational level; annual household income; alcohol intake; smoking status; morning sickness; questionnaires administered in early pregnancy; questionnaires administered in mid/late pregnancy

### Changes in PCS and MCS scores of primiparas and multiparas from early pregnancy to mid/late pregnancy by number of miscarriages or stillbirths

In the analysis limited to primiparas (Table 4), the group with a history of 1 miscarriage or stillbirth showed a significantly positive association with PCS score (β = 0.29, 95% CI 0.04 to 0.54, *p* = 0.0249) compared with the group with no history. This group with a history of 1 miscarriage or stillbirth showed a significantly positive association with the PF and RP scores on the SF-8 compared with the group with no history (Supplementary Table 1).

**Table 4:** Results of linear mixed model analyses of PCS and MCS scores for primiparas

Effect	Level	Period	Crude		Adjusted^a^	
	NMS		PCS	MCS	PCS	MCS
			β(95%CI)	β(95%CI)	β(95%CI)	β(95%CI)
Period		Early	reference	reference	reference	reference
		Mid/late	**1.07 (0.98, 1.16)**	**3.36 (3.28, 3.45)**	**1.85(1.47, 2.23)**	**2.90 (2.53, 3.27)**
NMS	0	Early	44.91 (44.83, 44.99)	45.74 (45.66, 45.83)	reference	reference
	1	Early	** − 0.71 (− 0.96, − 0.47)**	0.05 (− 0.19, 0.28)	** − 0.29 (− 0.52, − 0.06)**	0.02 (− 0.21, 0.24)
	2	Early	** − 1.11 (− 1.69, − 0.53)**	− 0.30 (− 0.86, 0.26)	− 0.43 (− 0.98, 0.12)	− 0.39 (− 0.94, 0.17)
	≥ 3	Early	** − 1.63 (− 2.79, − 0.47)**	− 0.78 (− 1.87, 0.31)	− 0.72 (− 1.85, 0.41)	− 0.96 (− 2.00, 0.08)
NMS × Period	0 ×	Mid/late	reference	reference	reference	reference
	1 ×	Mid/late	**0.53 (0.27, 0.79)**	0.21 (− 0.04, 0.46)	**0.29 (0.04, 0.54)**	0.13 (− 0.12, 0.37)
	2 ×	Mid/late	**0.68 (0.07, 1.30)**	0.20 (− 0.39, 0.80)	0.34 (− 0.26, 0.93)	0.09 (− 0.50, 0.69)
	≥ 3 ×	Mid/late	0.94 (− 0.21, 2.08)	0.25 (− 1.05, 1.55)	0.52 (− 0.60, 1.65)	0.16 (− 1.12, 1.44)

Boldface indicates significance at *p* values less than 0.05

*NMS* Number of miscarriages or stillbirths, *PCS* Physical Component Summary, *MCS* Mental Component Summary

^a^Adjusted for age during pregnancy; pre-pregnancy BMI; parity; physical activity; history of depression, anxiety disorder, dysautonomia, or schizophrenia; history of any physical disease; marital status; employed during early pregnancy; highest educational level; annual household income; alcohol intake; smoking status; morning sickness; questionnaires administered in early pregnancy; questionnaires administered in mid/late pregnancy

In the analysis limited to multiparas (Table 5), the group with ≥ 3 miscarriages or stillbirths showed a significantly positive association with PCS score (β = 0.81, 95% CI 0.14 to 1.48, *p* = 0.0183) compared with the group with no history. This group with a history of 1 miscarriage or stillbirth showed a significantly positive association with the RP and VT scores compared with the group with no history (Supplementary Table 2). In addition, the group with ≥ 3 miscarriages or stillbirths showed a significantly positive association with the PF and BP scores compared with the group with no history.

**Table 5:** Results of linear mixed model analyses of PCS and MCS scores for multiparas

Effect	Level	Period	Crude		Adjusted^a^	
	NMS		PCS	MCS	PCS	MCS
			β(95%CI)	β(95%CI)	β(95%CI)	β(95%CI)
Period		Early	reference	reference	reference	reference
		Mid/late	**0.29 (0.20, 0.37)**	**2.77 (2.69, 2.85)**	**0.74 (0.40, 1.09)**	**2.27 (1.94, 2.59)**
NMS	0	Early	45.32 (45.25, 45.40)	46.33 (46.25, 46.40)	reference	reference
	1	Early	** − 0.49 (− 0.67, − 0.32)**	** − 0.23 (− 0.41, − 0.06)**	** − 0.28 (− 0.45, − 0.11)**	** − 0.18 (− 0.35, − 0.01)**
	2	Early	** − 0.78 (− 1.12, − 0.44)**	− 0.20 (− 0.53, 0.13)	** − 0.45 (− 0.77, − 0.12)**	− 0.05 (− 0.36, 0.27)
	≥ 3	Early	** − 1.46 (− 2.09, − 0.83)**	− 0.10 (− 0.75, 0.54)	** − 0.90 (− 1.48, − 0.31)**	0.18 (− 0.43, 0.79)
NMS × Period	0 ×	Mid/late	reference	reference	reference	reference
	1 ×	Mid/late	0.18 (− 0.01, 0.37)	0.14 (− 0.04, 0.32)	0.17 (− 0.02, 0.36)	0.11 (− 0.07, 0.29)
	2 ×	Mid/late	0.21 (− 0.16, 0.58)	− 0.11 (− 0.45, 0.24)	0.15 (− 0.21, 0.51)	− 0.16 (− 0.50, 0.18)
	≥ 3 ×	Mid/late	**0.99 (0.29, 1.69)**	0.00 (− 0.69, 0.68)	**0.81 (0.14, 1.48)**	− 0.08 (− 0.76, 0.60)

Boldface indicates significance at *p* values less than 0.05

*NMS* Number of miscarriages or stillbirths, *PCS* Physical Component Summary, *MCS* Mental Component Summary

^a^Adjusted for age during pregnancy; pre-pregnancy BMI; parity; physical activity; history of depression, anxiety disorder, dysautonomia, or schizophrenia; history of any physical disease; marital status; employed during early pregnancy; highest educational level; annual household income; alcohol intake; smoking status; morning sickness; questionnaires administered in early pregnancy; questionnaires administered in mid/late pregnancy

### Differences in PCS and MCS scores of primiparas and multiparas in early pregnancy by number of miscarriages or stillbirths

As shown in Table 4, PCS scores of primiparas in early pregnancy were significantly lower in the groups with a history of 1 miscarriage or stillbirth (mean PCS = 44.23, β =  − 0.29, 95% CI − 0.52 to − 0.06, *p* = 0.0132) compared with the group with no history of miscarriage or stillbirth (mean PCS = 44.52).

As shown in Table 5, PCS scores of multiparas in early pregnancy were significantly lower in the groups with a history of 1 miscarriage or stillbirth (mean PCS = 45.42, β =  − 0.28, 95% CI − 0.45 to − 0.11, *p* = 0.0012), 2 miscarriages or stillbirths (mean PCS = 45.25, β =  − 0.45, 95% CI − 0.77 to − 0.12, *p* = 0.0073), and ≥ 3 miscarriages or stillbirths (mean PCS = 44.80, β =  − 0.90, 95% CI − 1.48 to − 0.31, *p* = 0.0026) compared with the group with no history of miscarriage or stillbirth (mean PCS = 45.70). MCS score was significantly lower in the group with a history of 1 miscarriage or stillbirth (mean MCS = 46.79, β =  − 0.18, 95% CI − 0.35 to − 0.01, *p* = 0.0372) compared with the group with no history of miscarriage or stillbirth (mean MCS = 46.97).

## Discussion

The three main results of this study were as follows: 1) a lower PCS score in early pregnancy was associated with a more frequent history of miscarriage or stillbirth, as were lower SF-8 subscale scores, especially for PF, RP, and SF; 2) PCS score significantly increased during pregnancy in pregnant women with either 1 or ≥ 3 miscarriages or stillbirths compared with those with no history of miscarriage or stillbirth; and 3) in multiparas, a low MCS score in early pregnancy was associated with history of miscarriage or stillbirth.

### Association of lower PCS score and lower PF, RP, and SF subscale scores in early pregnancy with more previous miscarriages and stillbirths

The present finding that pregnant women with a more frequent history of miscarriages and stillbirths had a lower PCS score in early pregnancy is similar to that of previous studies [18, 19]. Pregnant women with a history of miscarriage or stillbirth have a fear of miscarriage or stillbirth in the subsequent pregnancy, which increases anxiety and depression [15]. As the number of previous miscarriages and stillbirths increases, the more fear and anxiety accumulates and the more QOL during pregnancy is affected. On the SF-8, the PF, RP, and SF scores were found to be lower with a more frequent history of miscarriage or stillbirth in this study. These subscales ask to what extent physical reasons interfered with the respondents’ daily lives, tasks, and social roles. Pregnant women with RPL are sensitive to physical symptoms reminiscent of miscarriage (e.g., heaviness in the lower abdomen, increased virginal discharge reminiscent of bleeding, and loss of morning sickness) in the early stages of pregnancy due to anxiety and fear of miscarriage, and they tend to rest as much as possible and refrain from going out [27]. This suggests that pregnant women with a history of miscarriage or stillbirth experience decreased physical activity in early pregnancy, which interferes with their daily activities and decreases their PCS score.

### Significantly increased PCS score during pregnancy in women with 1 or ≥ 3 previous miscarriages and stillbirths compared with those with no history of miscarriage or stillbirth

In this study, the SF-8 scores for both PCS and MCS increased from early pregnancy to mid/late pregnancy. The PCS significantly increased during pregnancy in the groups with either 1 or ≥ 3 miscarriages or stillbirths compared with the group with no miscarriage or stillbirth. The slope of the increase was steeper when the analysis was restricted to primiparas. It has been suggested that women with RPL develop severe depression and anxiety in the early stages of pregnancy, but this lessens during the pregnancy because the fetus grows beyond the period of miscarriage risk and they feel more secure about their pregnancy [16]. Similarly, in the present study, we speculate that the PCS score of pregnant women with a history of miscarriage or stillbirth may have increased during mid/late pregnancy due to lessened fear and anxiety about miscarriage compared with in early pregnancy and due to increased physical activity. In a longitudinal study that examined pregnant women’s QOL using the SF-36 [28–30], PCS score decreased during pregnancy, and Chang et al. [28] argued that changes in pregnant women’s body shape and weight gain during pregnancy affected the decline in PCS score during mid/late pregnancy. However, in the present study, PCS score increased regardless of the presence or absence of a history of miscarriage or stillbirth, which is a new finding. Bahadoran et al. [31] found that physical activity and social support were negatively correlated in a study of pregnant women in the third trimester. In Japan, there is a tradition (called *Satogaeri*) in which pregnant women move to their parents’ home during mid/late pregnancy to receive support from their parents. We considered this tradition as one possible reason for this association of PCS score and QOL in pregnant women. In this study, MCS score also increased during pregnancy, similar to previous studies [28, 29].

### Association of lower MCS score of multiparas in early pregnancy with more previous miscarriages and stillbirths

In the analysis limited to multiparas, it was found that pregnant women with a history of 1 miscarriage or stillbirth had a lower MCS score in early pregnancy. Since depression in early pregnancy decreases the QOL of pregnant women [17], pregnant women with a history of miscarriage or stillbirth experience more depression in early pregnancy [15, 16], so it is possible this is why the present study also found decreased MCS scores. Previous studies have reported that primiparas are more anxious and fearful in pregnancy after miscarriage than in multiparas who have live babies [12, 32]. In the present study, we did not compare the MCS scores of primiparas and multiparas. However, there was a significant difference in this score in multiparas in early pregnancy. We speculate that multiparas with a history of miscarriage or stillbirth may feel stressed by childcare in the early pregnancy.

The more frequent the history of miscarriage or stillbirth, the lower a pregnant woman’s PCS score in early pregnancy, indicating a need for the support of pregnant women with a history of miscarriage or stillbirth. Pregnant women with a history of miscarriage or stillbirth may be excessively sedentary and less physically active in early pregnancy for fear of miscarriage or stillbirth. However, there is no evidence that rest prevents miscarriage [33, 34], and it is difficult to say that resting to the extent that it reduces QOL is an appropriate way to spend pregnancy. Therefore, in the early stages of pregnancy for women with a history of miscarriage or stillbirth, in addition to psychological support, it would be desirable to provide support that includes a detailed assessment of the physical activities of daily living to ascertain whether excessive rest is interfering with daily living. Even if QOL is low in early pregnancy, it increases toward mid/late pregnancy, more so than for pregnant women with no history of miscarriage or stillbirth. This finding is encouraging and hopeful for pregnant women with a history of miscarriage or stillbirth.

The strengths of this study include its sample size, with the JECS being a cohort study with more than 100,000 participants, and a low level of missing data or dropouts (about 84% of participants remain).

The limitations of this study and future work include the following. Although various factors were included as covariates in the generalized linear mixed model analysis, other factors related to pregnant women’s QOL may not have been included. In the present study, the QOL of study participants increased during pregnancy, which is a new result that differs from that of previous studies. However, the second time point of the survey was at about 27 weeks of gestation, so it cannot be said that the changes encompassed the last trimester of pregnancy or that the trimesters were strictly defined. In addition, future studies should compare QOL between pregnant women, including those with a history of miscarriage or stillbirth, and the fathers.

## Conclusions

A cohort study of 82,013 pregnant women using JECS data revealed that women with a history of miscarriage or stillbirth had a lower PCS score in early pregnancy. However, compared with pregnant women with no history of miscarriage or stillbirth, women with a history of miscarriage or stillbirth showed higher PCS scores in mid/late pregnancy. These findings suggest that healthcare professionals need to pay close attention to whether daily life in the early stages of pregnancy is being negatively affected in pregnant women with a history of miscarriage or stillbirth.

## Supplementary Information

**Additional file 1: Supplementary Table 1.** Results of linear mixed model analyses of SF-8 subscale scores for primiparas. **Supplementary Table 2.** Results of linear mixed model analyses of SF-8 subscale scores for multiparas.

## Data Availability

Data are unsuitable for public deposition due to ethical restrictions and the legal framework of Japan. It is prohibited by the Act on the Protection of Personal Information (Act No. 57 of 30 May 2003, amendment on 9 September 2015) to publicly deposit data containing personal information. The Ethical Guidelines for Medical and Health Research Involving Human Subjects enforced by the Japan Ministry of Education, Culture, Sports, Science, and Technology and the Ministry of Health, Labour and Welfare also restrict the open sharing of epidemiological data. All inquiries about access to data should be sent to: jecs-en@nies.go.jp. The person responsible for handling enquiries sent to this e-mail address is Dr Shoji F. Nakayama, JECS Programme Office, National Institute for Environmental Studies.

## Abbreviations

JECS: Japan Environment and Children’s Study
RPL: Recurrent pregnancy loss
PCS: Physical Component Summary
MCS: Mental Component Summary
QOL: Quality of life
SF-8: 8-Item Short-Form Health Survey
SF-36: 36-Item Short-Form Health Survey
GH: General health
PF: Physical functioning
RP: Role physical
BP: Bodily pain
VT: Vitality
SF: Social functioning
MH: Mental health
RE: Role emotional
