# Supplementary material for: Longitudinal study of the relationship between number of prior miscarriages or stillbirths and changes in quality of life of pregnant women: the Japan Environment and Children’s Study (JECS)
Source: BMC Pregnancy Childbirth. 2023 Apr 28;23:297. doi: 10.1186/s12884-023-05578-6 (PMC10148530; doi:10.1186/s12884-023-05578-6)
Supplement: Supplementary file 1 — Additional file 1: Supplementary Table 1. Results of linear mixed model analyses of SF-8 subscale scores for primiparas. Supplementary Table 2. Results of linear mixed model analyses of SF-8 subscale scores for multiparas. [file 12884_2023_5578_MOESM1_ESM.docx]

| **Supplementary Table 1** Results of linear mixed model analyses of SF-8 subscale scores for primiparas | | | | | | | | | | |
| --- | --- | --- | --- | --- | --- | --- | --- | --- | --- | --- |
| Effect | Level |  | Adjusted^a^ |  |  |  |  |  |  |  |
|  | NMS | Period | General health | Physical functioning | Role physical | Bodily pain | Vitality | Social functioning | Mental health | Role emotional |
|  |  |  | β(95%CI） | β(95%CI） | β(95%CI） | β(95%CI） | β(95%CI） | β(95%CI） | β(95%CI） | β(95%CI） |
| Period | —— | Early | reference | reference | reference | reference | reference | reference | reference | reference |
|  | —— | Mid/late | **5.57**  **(5.19, 5.96)** | 0.16  (−0.26, 0.57) | **4.22**  **(3.74, 4.70)** | **−2.04**  **(−2.48, −1.60)** | **4.36**  **(3.99, 4.73)** | **3.40**  **(2.91, 3.89)** | **1.05**  **(0.70, 1.41)** | **2.68**  **(2.25, 3.12)** |
| NMS | 0 | Early | reference | reference | reference | reference | reference | reference | reference | reference |
|  | 1 | Early | **0.30 (0.08, 0.53)** | **−0.55 (−0.81, −0.30)** | **−0.60 (−0.89, −0.31)** | 0.16 (−0.09, 0.41) | 0.06(0.16, 0.29) | **−0.32 (−0.62, −0.02)** | −0.05 (−0.27, 0.16) | −0.12 (−0.39, 0.15) |
|  | 2 | Early | 0.38 (−0.17, 0.93) | **−0.82 (−1.42, −0.22)** | **−1.11 (−1.82, −0.39)** | 0.19 (−0.38, 0.76) | −0.16 (−0.70, 0.38) | **−1.05 (−1.77, −0.33)** | −0.45 (−0.96, −0.07) | −0.29 (−0.95, 0.36) |
|  | ≥3 | Early | 0.10 (−0.90, 1.10) | **−1.50 (−2.78, −0.22)** | **−1.81 (−3.31, −0.32)** | 0.65 (−0.35, 1.66) | −0.32 (−1.28, 0.64) | **−1.94 (−3.22, −0.65)** | −0.39 (−1.32, 0.54) | **−1.51 (−2.94, −0.77)** |
| NMS × Period | 0 × | Mid/late | reference | reference | reference | reference | reference | reference | reference | reference |
|  | 1 × | Mid/late | −0.03 (−0.28, 0.22) | **0.41 (0.13, 0.70)** | **0.57 (0.25, 0.89)** | −0.07 (−0.36, 0.22) | 0.20 (−0.04, 0.44) | 0.29 (−0.03, 0.61) | 0.15 (−0.09, 0.38) | 0.22 (−0.07, 0.51) |
|  | 2 × | Mid/late | −0.48 (−1.06, 0.11) | 0.47 (−0.22, 1.15) | 0.60 (−0.19, 1.39) | 0.22 (−0.40, 0.85) | 0.09 (−0.47, 0.65) | **0.89 (0.08, 1.70)** | 0.22 (−0.33, 0.76) | −0.16 (−0.92, 0.60) |
|  | ≥3 × | Mid/late | −0.05 (−1.02, 0.92) | 0.98 (−0.31, 2.26) | 0.88 (−0.68, 2.45) | −0.17 (−1.36, 1.03) | −0.26 (−1.28, 0.76) | 0.70 (−0.81, 2.22) | 0.04 (−1.01, 1.08) | 0.71 (−0.94, 2.36) |
| NMS=number of miscarriages or stillbirths, Boldface indicates significance at p values less than 0.05  ^a^ Adjusted for age during pregnancy; pre-pregnancy BMI; parity; physical activity; history of depression, anxiety disorder, dysautonomia, or schizophrenia; history of any physical disease; marital status; employed during early pregnancy; highest educational level; annual household income; alcohol intake; smoking status; morning sickness; questionnaires administered in early pregnancy; questionnaires administered in mid/late pregnancy. | | | | | | | | | | |

| **Supplementary Table 2** Results of linear mixed model analyses of SF-8 subscale scores for multiparas | | | | | | | | | | |
| --- | --- | --- | --- | --- | --- | --- | --- | --- | --- | --- |
| Effect | Level |  | Adjusted^a^ |  |  |  |  |  |  |  |
|  | NMS | Period | General health | Physical functioning | Role physical | Bodily pain | Vitality | Social functioning | Mental health | Role emotional |
|  |  |  | β(95%CI） | β(95%CI） | β(95%CI） | β(95%CI） | β(95%CI） | β(95%CI） | β(95%CI） | β(95%CI） |
| Period | —— | Early | reference | reference | reference | reference | reference | reference | reference | reference |
|  | —— | Mid/late | **4.44**  **(4.08, 4.80)** | −0.26  (−0.63, 0.11) | **2.85**  **(2.43, 3.28)** | **−3.25**  **(−3.66, −2.84)** | **3.49**  **(3.15, 3.84)** | **2.32**  **(1.88, 2.76)** | **0.55**  **(0.24, 0.87)** | **1.69**  **(1.32, 2.05)** |
| NMS | 0 | Early | reference | reference | reference | reference | reference | reference | reference | reference |
|  | 1 | Early | −0.01 (−0.18, 0.17) | **−0.34 (−0.52, −0.16)** | **−0.44 (−0.65, −0.23)** | −0.17 (−0.37, 0.03) | −0.16(−0.33, 0.01) | **−0.45 (−0.67, −0.24)** | **−0.24 (−0.40, −0.07)** | −0.14 (−0.32, 0.04) |
|  | 2 | Early | 0.01 (−0.31, 0.32) | **−0.66 (−1.01, −0.30)** | **−0.59 (−1.00, −0.18)** | −0.20 (−0.57, 0.17) | 0.04 (−0.28, 0.37) | −0.37 (−0.79, 0.04) | −0.29 (−0.59, 0.02) | −0.11 (−0.47, 0.24) |
|  | ≥3 | Early | 0.18 (−0.44, 0.79) | **−0.94 (−1.59, −0.29)** | **−0.82 (−1.58, −0.05)** | **−0.93 (−1.64, −0.21)** | 0.13 (−0.47, 0.73) | −0.57 (−1.36, 0.21) | 0.06 (−0.51, 0.63) | −0.45 (−1.16, 0.27) |
| NMS × Period | 0 × | Mid/late | reference | reference | reference | reference | reference | reference | reference | reference |
|  | 1 × | Mid/late | 0.07 (−0.12, 0.26) | 0.18 (−0.02, 0.39) | **0.30 (0.07, 0.53)** | 0.02 (−0.19, 0.24) | **0.21 (0.02, 0.39)** | 0.17 (−0.07, 0.41) | 0.13 (−0.04, 0.31) | 0.10 (−0.10, 0.30) |
|  | 2 × | Mid/late | −0.05 (−0.40, 0.31) | 0.33 (−0.07, 0.74) | −0.01 (−0.47, 0.45) | 0.10 (−0.30, 0.50) | −0.03 (−0.39, 0.32) | −0.18 (−0.64, 0.27) | 0.12 (−0.21, 0.45) | −0.22 (−0.61, 0.18) |
|  | ≥3 × | Mid/late | 0.10 (−0.58, 0.79) | **0.88 (0.10, 1.66)** | 0.52 (−0.33, 1.38) | **1.21 (0.46, 1.97)** | −0.10 (−0.73, 0.53) | 0.03 (−0.86, 0.92) | 0.34 (−1.27, 0.94) | 0.46 (−0.37, 1.28) |
| NMS=number of miscarriages or stillbirths, Boldface indicates significance at p values less than 0.05  ^a^ Adjusted for age during pregnancy; pre-pregnancy BMI; parity; physical activity; history of depression, anxiety disorder, dysautonomia, or schizophrenia; history of any physical disease; marital status; employed during early pregnancy; highest educational level; annual household income; alcohol intake; smoking status; morning sickness; questionnaires administered in early pregnancy; questionnaires administered in mid/late pregnancy. | | | | | | | | | | |
